# Factors influencing intra-group data sharing in China’s central state-owned enterprises: An information ecology perspective

**DOI:** 10.1371/journal.pone.0343558

**Published:** 2026-02-20

**Authors:** Dongxu Wang, Xifeng Ning

**Affiliations:** 1 School of Economics and Management, Beijing Jiaotong University, Beijing, China; 2 China National Medical Device CO., LTD, Beijing, China; 3 PetroChina Planning and Engineering Institute, Beijing, China; Sichuan Agricultural University, CHINA

## Abstract

**Objectives:**

This paper aims to clarify the factors influencing intra-group data sharing within large central state-owned enterprises (CSOEs) in China, thereby promoting cross-organizational data circulation and sharing.

**Methods:**

Based on the Stimulus-Organism-Response (SOR) theoretical framework, and integrating Information Ecology Theory and the Theory of Planned Behavior (TPB), this paper develops a research model examining the factors influencing data sharing within CSOEs. Data were collected through a survey questionnaire, yielding 478 valid responses from employees across various CSOEs. Structural Equation Modeling (SEM) was employed to empirically test the research hypotheses.

**Results:**

(1) Inter-organizational relationship strength and employee data literacy positively influenced both attitude toward data sharing and data sharing intention. (2) Data quality positively influenced intention but had no significant effect on attitude. (3) Data culture and institutional norms exerted a positive influence on both attitude and intention, with institutional norms showing a stronger effect. (4) Data platform integration capability directly influenced data sharing behavior, while platform security assurance capabilities enhanced both intention and behavior. (5) Data sharing intention positively influenced data sharing behavior, but attitude toward data sharing had no significant effect on data sharing intention.

**Conclusions:**

This study identifies a “Cognitive-Intention Decoupling” phenomenon, and defines the boundary conditions of TPB in the context of strong administrative constraints. It reveals an “Adaptive Reconstruction” mechanism of attitude under strong administrative directives, where organizational normative pressure suppresses the traditional pathway of building internal attitudes based on technological perceptions. Furthermore, the study elucidates the differentiated roles of technological features, finding that platform integration capability acts as a “behavioral shortcut” to bypass intention and directly drive behavior, while data security assurance capability serves as a critical hygiene factor stimulating both intention and behavior.

## 1 Introduction

With the rapid development of advanced digital technologies, modern society has entered the digital economy era [1]. Data, recognized as the fifth major production factor following land, labor, capital, and technology, has become a critical engine driving socioeconomic development [2]. The Chinese government attaches great importance to the role of data factors in economic growth. As key pillars of the national economy, CSOEs serve as the core force in implementing the national data factor development strategy. Intra-group data sharing is a crucial means to unlock the value of their data assets. However, due to their diversified business structures and hierarchical organizational systems, intra-group data sharing scenarios in CSOEs are complex and involve numerous influencing factors. Consequently, data sharing initiatives often encounter challenges such as “unwillingness, hesitation, and incapability” during the data sharing process [3]. Systematically analyzing the influencing factors and driving mechanisms of data sharing within CSOEs is key to breaking down barriers to cross-organizational data sharing. It is of great significance for unlocking the value of data and enhancing enterprise operational and management efficiency.

Academia has extensively explored the topic of data sharing, but most studies focus on single organizational attributes, making it difficult to fully explain CSOEs with diverse organizational characteristics. Existing literature can be broadly categorized into three perspectives: (1) the administrative perspective [4,5], which focuses on data sharing among government departments, emphasizing the role of organizational governance and administrative directives; (2) the market perspective [6,7], which centers on data sharing among supply chain enterprises, highlighting reciprocal mechanisms and economic incentives; and (3) the professional perspective [8–11], which examines data sharing norms and individual perceptions in fields such as healthcare and scientific research. However, CSOEs possess dual characteristics of market-oriented operations and administrative control, characterized by multi-level legal structures, complex industrial chains, and high data sensitivity. Consequently, a significant mismatch exists between current theoretical frameworks and the operational realities of CSOEs. Governmental data sharing theories often overlook market incentive demands, while purely commercial supply chain data sharing theories fail to account for administrative constraints. Additionally, data sharing theories from specialized fields like healthcare and scientific research often lack generalizability to the corporate context. Within the complex context of CSOEs operating in a “semi-administrative, semi-market” environment, there remains a lack of a systematic theoretical framework for interpreting their data sharing behaviors.

This paper addresses the following issues: First, how to develop a theoretical framework for the complex behavior of intra-group data sharing in CSOEs? Second, what are the key influencing factors in this context? Third, what are the driving mechanisms and path relationships among these factors? To this end, this paper adopts the SOR theoretical model as its foundation, synthesizes the influencing factors based on Information Ecology Theory, and incorporates TPB to analyze data sharing processes. It develops a research model of the factors influencing data sharing within CSOEs and empirically tests it.

## 2 Related work and integrated framework

### 2.1 Related work

Data sharing refers to the interoperability and collaborative utilization of data between different entities through systematic mechanisms, encompassing interactions among individuals, cross-regional organizations, and multi-level institutions [12]. In terms of research on the factors influencing data sharing, Vest [13] employed a cross-sectional analysis to examine the factors influencing data sharing between U.S. local and state health agencies, identifying information system capabilities and organizational governance models as key factors. In addition, legal policies, data standardization, and cultural factors have been found to influence data sharing. Xu [14] analyzed the key factors influencing scientific data sharing based on the fuzzy-DEMATEL method, identifying 16 key factors, including data sharing policies, platforms, institutional culture, researchers’ intentions, trust, and data quality. Based on TPB, Kim developed research models on the influencing factors of data sharing among STEM researchers [10] and internet researchers [11], and used SEM to validate the influence of attitudinal beliefs, disciplinary norms, social norms, data warehouse, and reuse experiences on attitudes, intentions, and behaviors toward data sharing. Although there is a wealth of research on data sharing, there is still a gap in research on data sharing within CSOEs.

### 2.2 Integrated framework

CSOEs generally refer to enterprises directly supervised by the State-owned Assets Supervision and Administration Commission (SASAC) [15], which typically operate as large conglomerates. In the context of data factorization, data sharing is a collaborative activity involving multi-stakeholder ownership allocation. This paper defines data sharing in CSOEs as follows: departments and subsidiaries within CSOEs exchange and reuse cross-organizational data through digital tools to meet business and managerial needs, while adhering to corporate institutional norms. To uncover the complex driving forces behind data sharing within CSOEs, this study constructs a hierarchical theoretical framework. This framework adopts the SOR model as its overarching logic, and integrates both Information Ecology Theory and the TPB.

#### 2.2.1 The overarching logic: SOR framework.

SOR theory is a classical theoretical model in behavioral science [16], elucidating the pathway through which environmental stimuli influence an individual’s internal psychological state, ultimately leading to a behavioral response. In the context of data sharing within CSOEs, data sharing is not merely a technical operation but rather a behavioral decision made by organizational members in response to specific environmental stimuli. Therefore, this study adopts the SOR model as the overarching framework to characterize the complex process ranging from environmental stimuli and individual psychological changes to the final occurrence of data sharing behavior.

#### 2.2.2 The taxonomy of stimuli: Information ecology perspective.

Within the SOR model, “Stimulus” typically refers to external environmental triggers that elicit individual responses. However, given the complex internal structure of CSOEs, the selection of “stimuli” may appear fragmented and lack theoretical grounding without a systematic classification taxonomy. Information Ecology Theory [17], an interdisciplinary field integrating information science, ecology, and management, uses ecological metaphors to reveal the complex synergistic relationships among humans, technologies, and environments in information activities. Therefore, this study introduces Information Ecology Theory as the taxonomic foundation for defining stimulus sources, utilizing its four core factors—Information Person, Information Resource, Information Environment, and Information Technology—to systematically categorize the external factors influencing data sharing.

#### 2.2.3 The cognitive mechanism: Theory of planned behavior.

TPB is used to explain and predict the behavioral intentions and actual behaviors of individuals or groups in specific contexts [18]. It posits that behavioral intention is the primary determinant of behavior, which is determined by attitude toward the behavior, subjective norms, and perceived behavioral control [19]. This study integrates TPB to open the black box of the “Organism” component within the SOR model, serving as the core mechanism for explaining cognitive transformation. We map TPB’s core constructs—”attitude” and “intention”—to the “Organism” element in SOR, representing individuals’ psychological evaluation and state of readiness to respond to external ecological stimuli. Meanwhile, “behavior” is mapped to the “Response” component.

#### 2.2.4 Theoretical integration and logical hierarchy.

**Fig 1:**
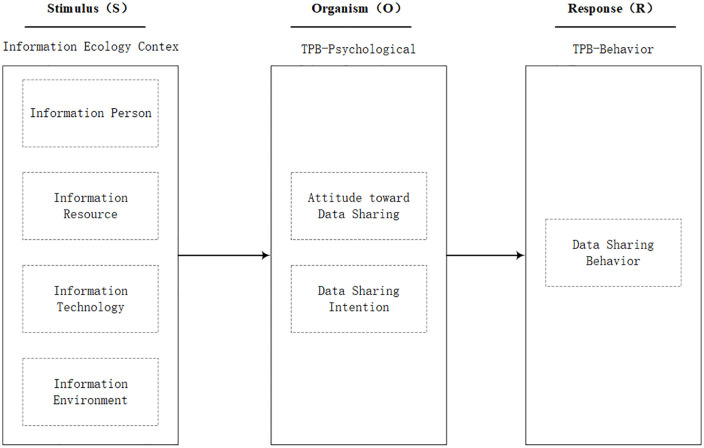
Conceptual model of factors influencing data sharing within CSOEs.

In summary, this study constructs a hierarchical conceptual model, as shown in Fig 1. This framework is characterized by the following two dimensions:

**Logical Hierarchy**: The variables defined by Information Ecology Theory constitute the objective external context (Stimulus), while the variables from TPB—attitude toward data sharing and data sharing intention—constitute the subjective psychological processes (Organism), and the TPB variable—behavior—constitutes the behavioral response (Response).

**Causal Mechanism**: The model follows a logical chain where “the ecological environment drives cognitive evaluation, and cognitive evaluation drives behavioral response.” The four factors of information ecology serve as antecedent variables, which influence employees’ attitudes toward data sharing and data sharing intentions, ultimately driving the occurrence of data sharing behavior.

## 3 Hypotheses development

Based on the integrated framework proposed in Section 2, this section develops hypotheses following the S-O-R logic.

### 3.1 Stimulus: Information ecology factors

#### 3.1.1 Information person.

In the context of data sharing within CSOEs, factors related to the Information Person dimension serve as the primary stimulus.

**Relationship strength:** Relationship strength reflects the degree of trust and reciprocity between organizations [20]. In high-trust relationships, organizations are more likely to believe that partners will not misuse shared data [21], thereby leading to a positive evaluation of data sharing. Ahmad [22] demonstrated that trust has a significant positive influence on information sharing. Reciprocity is another characteristic of strong relationships [23], organizations believe that data sharing will bring rewards from partners in strong relationships, thereby forming positive attitudes and intentions to data sharing [24]. Li [21] found that strong social capital relationships significantly enhance high-quality information sharing in Chinese organizational contexts. Based on the above analysis, this paper proposes the following hypotheses:

H1a/b. Relationship strength between organizations positively influences (a) attitude toward data sharing and (b) data sharing intention.

**Employee data literacy:** Data literacy refers to an individual’s ability to understand, interpret, and apply data [25], directly influencing the efficiency of team collaboration and organizational innovation [26]. Knowledge Management Theory argues that an individual’s level of knowledge and skills directly influences their attitudes toward knowledge sharing. As a specific combination of knowledge and skills in a digital environment, data literacy directly influences the organism’s cognitive evaluation, specifically employees’ attitudes toward data sharing. Furthermore, employees with high data literacy can understand and process data more effectively, thereby being more willing to enhance the organization’s overall performance through data sharing. Based on the above analysis, this paper proposes the following hypotheses:

H2a/b. Employee data literacy positively influences (a) attitude toward data sharing and (b) data sharing intention.

#### 3.1.2 Information resource.

**Data quality:** Data quality refers to the extent to which data meets the requirements of a specific application scenario [27,28]. According to TPB, attitudes stem from evaluations of behavioral outcomes. When employees perceive the data to be shared as high-quality, they are likely to believe that data sharing can generate value. Driven by this resource stimulus, this expectation of positive outcomes translates into favorable cognitive evaluations of data sharing behavior. Wu [29] found that in supply chain management, high-quality data enhances member firms’ attitudes toward information sharing and collaboration. In the context of scientific data sharing, Kim [11] found that high-quality data makes it easier for researchers to anticipate positive results, thereby stimulating the organism’s data sharing intentions. Based on the above analysis, this paper proposes the following hypotheses:

H3a/b. Data quality positively influences (a) attitude toward data sharing and (b) data sharing intention.

#### 3.1.3 Information environment.

**Data culture:** Data culture refers to the consensus and behavioral norms formed within an enterprise or organization regarding the value, management, and application of data [30]. It exerts a subtle and gradual influence on employees’ psychological cognition, constituting a form of “soft” ideological stimulus. Through the cognitive mechanism described by Organizational Culture Theory, this stimulus has a profound influence on employees’ cognitive attitudes [31]. Additionally, Strategic Alignment Theory suggests that clear strategic direction unifies organizational actions, thereby enhancing the organism’s attitudes and intentions toward related behaviors [32]. Faniel [33] found that a positive data culture encourages organizations and researchers to view data as a shareable resource, thereby encouraging data owners to openly share their data with others. Based on the above analysis, this paper proposes the following hypotheses:

H4a/b. Data culture positively influences (a) attitude toward data sharing and (b) data sharing intention.

**Institutional norms:** Institutional norms refer to the policies, regulations, and behavioral guidelines established within an organization. Clear institutional norms provide a legal basis and behavioral boundaries for data sharing, constituting a form of “hard” regulatory stimulus. Well-defined responsibilities, authorities, and incentive policies enhance employees’ perceived behavioral control, thereby improving their cognitive evaluation of data sharing. Through the mechanism described by Social Norm Theory, individuals’ attitudes are influenced by social norms and group expectations. A clear data sharing policy makes employees perceive data sharing as the organization’s standard behavior, thereby fostering positive cognitive attitudes toward data sharing. When employees recognize that data sharing is a behavior advocated and supported by the organization, their data sharing intention also increases. Waithira [34] pointed out that establishing an organization’s data management policy is the first step in promoting data sharing. Based on the above analysis, this paper proposes the following hypotheses:

H5a/b. Institutional norms positively influence (a) attitude toward data sharing and (b) data sharing intention.

#### 3.1.4 Information technology.

The data platform, as an information technology tool that supports data sharing, constitutes a crucial form of technological stimulus.

Data platform integration capability is reflected in its ability to achieve integration and collaboration across diverse systems, technical architectures, and data sources [35]. On the one hand, acting as a facilitating stimulus, a high level of data integration capability can significantly lower the technical barriers to data interaction. According to the perceived behavioral control logic of TPB, when employees perceive data sharing as an easily accomplished task, their data sharing intention increases. On the other hand, based on the mechanism of Task-Technology Fit (TTF) theory, when technological characteristics align with task requirements, the actual use of the technology increases directly. As an adaptive technology, data platforms integration capability directly promotes data sharing behavior.

Data platform security assurance capability refers to the ability to prevent data leakage, tampering, and unauthorized access. Given the highly sensitive nature of data in CSOEs, security assurance capability constitutes a critical protective stimulus in employee decision-making. Robust platform security assurance capability significantly reduces employees’ perceived risk of data breach responsibility, thereby stimulating stronger data sharing intention. Furthermore, platform security assurance capability provides a technical safeguard that compensates for deficiencies in interpersonal trust. According to the mechanism of organizational trust theory, such systematic security protection not only enhances employees’ subjective intention but also provides the necessary safety guarantees for the actual occurrence of data sharing behavior. Based on the above analysis, this paper proposes the following hypotheses:

H6a/b. Data platform integration capability positively influences (a) data sharing intention and (b) data sharing behavior.

H7a/b. Data platform security assurance capability positively influences (a) data sharing intention and (b) data sharing behavior.

### 3.2 Organism and response: Attitude, intention and behavior

Attitude toward data sharing represents the organism’s cognitive evaluation of data sharing behavior, including their perceptions of the advantages, disadvantages, risks, and potential benefits of data sharing. Data sharing intention refers to the subjective willingness of data sharing entities toward data sharing behavior, reflecting the organism’s psychological readiness or intend to share data. Data sharing behavior refers to the actual behavioral response taken by data sharing entities in the process of sharing data, reflecting their choices in specific contexts. TPB posits that behavior is determined by behavioral intentions, with attitudes serving as an important factor influencing behavioral intentions. Meanwhile, behavioral intention plays a mediating role between attitude and behavior. Kim [10] confirmed the applicability of this theory in scientific data sharing research. Based on the above analysis, this paper proposes the following hypotheses:

H8. Attitude toward data sharing positively influences data sharing intention.

H9. Data sharing intention positively influences data sharing behavior.

H10. Data sharing intention plays a mediating role in the relationship between attitude toward data sharing and data sharing behavior.

**Fig 2:**
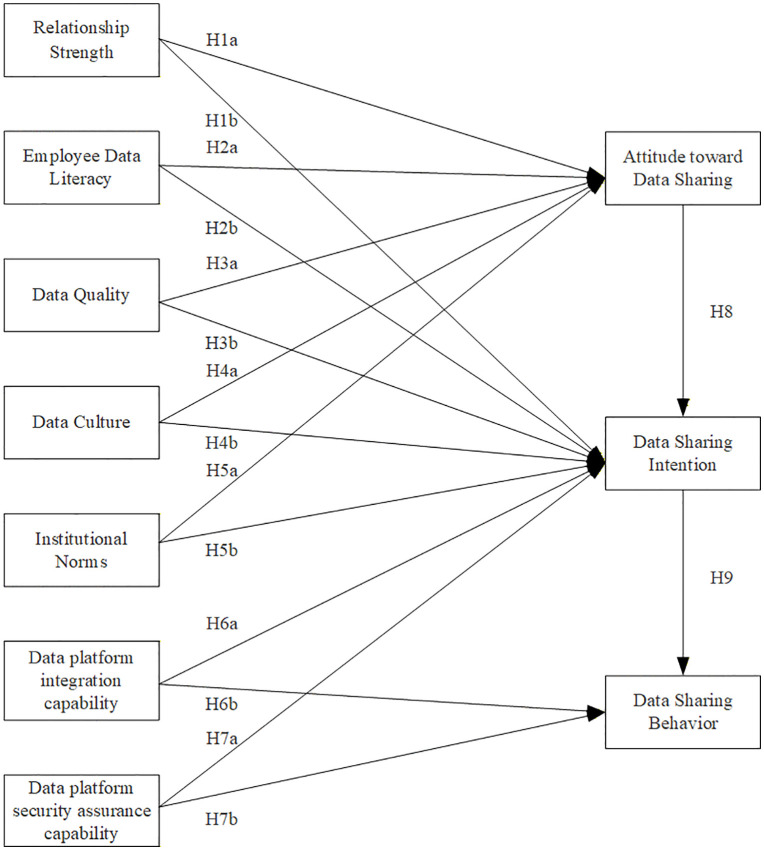
Research model of factors influencing data sharing within CSOEs.

Summary of research hypotheses as shown in Table 1. Based on the theoretical foundation and research hypotheses outlined above, a research model for data sharing within CSOEs has been developed, as shown in Fig 2.

**Table 1:** Summary of research hypotheses.

Target Construct (DV)	Hypothesis	Path (Relationship)	Predicted Direction
Antecedents of attitude toward data sharing	H1a	Relationship strength → Attitude toward data sharing	+
	H2a	Employee data literacy → Attitude toward data sharing	+
	H3a	Data quality → Attitude toward data sharing	+
	H4a	Data culture → Attitude toward data sharing	+
	H5a	Institutional norms → Attitude toward data sharing	+
Antecedents of data sharing intention	H1b	Relationship strength → Data sharing intention	+
	H2b	Employee data literacy → Data sharing intention	+
	H3b	Data quality → Data sharing intention	+
	H4b	Data culture → Data sharing intention	+
	H5b	Institutional norms → Data sharing intention	+
	H6a	Data platform integration capability → Data sharing intention	+
	H7a	Data platform security assurance capability → Data sharing intention	+
	H8	Attitude toward data sharing → Data sharing intention	+
Antecedents of data sharing behavior	H6b	Data platform integration capability → Data sharing behavior	+
	H7b	Data platform security assurance capability → Data sharing behavior	+
	H9	Data sharing intention → Data sharing behavior	+
Mediation Effects	H10	Attitude toward data sharing → Data sharing intention → Data sharing behavior	Mediated

## 4 Research design

This study adopted a research paradigm that combines theoretical construction and empirical testing. First, based on theoretical foundations and literature review, relationships among core variables were identified to establish the research model. Second, develop measurement items suitable for this paper’s research context by referring to established scales, and collect data through a survey questionnaire. Finally, data analysis and hypothesis verification were conducted using SEM.

### 4.1 Questionnaire design

The questionnaire design for this study consisted of three steps: First, by referencing established and widely validated scales from existing literature, combining them with the research context of this study, we developed an initial measurement scale for factors influencing data sharing within CSOEs. Second, invite four domain experts to review and revise the wording, grouping, and sequencing of the questionnaire items. Third, a pilot test was conducted on a small scale. After analyzing the reliability and validity of the pilot responses, unsuitable items were removed to finalize the questionnaire, as shown in S1 Table.

### 4.2 Sample selection and data collection

Given the broad scope of this study and aim to encompass multiple industries and organization types, implementing a random sampling survey posed significant challenges. Therefore, this study primarily engaged relevant organizations through resources such as alumni networks, colleagues, friends, and vendors serving CSOEs, followed by targeted redistribution of the questionnaire link using a snowball sampling strategy. A total of 582 questionnaires were collected in this survey. After removing invalid responses, 478 valid questionnaires were retained, yielding a valid response rate of 81.8%. The formal questionnaire consisted of 31 measurement items. The number of valid responses exceeded ten times the number of items, which met the recommended sample size requirements for SEM and satisfied the needs of this study [36]. Among the respondents, males accounted for 58.2%, slightly higher than females. The age range was primarily distributed between 26 and 45 years old, accounting for 87%. Additionally, nearly 80% held a master’s degree or higher. The surveyed companies were distributed across multiple industries, including mining and energy, infrastructure, defense manufacturing, information and communications, public services, and financial investments. Among the surveyed organizations, business units accounted for the largest proportion at 56.9%, followed by digital technology units, research units, and headquarters functional departments.

Informed consent was obtained from all subjects involved in the study. Prior to data collection, all participants were informed about the study’s objectives and the confidentiality of their data. Written informed consent was obtained electronically from all participants, who confirmed their agreement to participate before accessing the survey questions.

## 5 Data analysis

### 5.1 Measurement model testing

#### 5.1.1 Common method bias test.

Given that the data in this study were collected through self-reports at a single point in time, potential common method bias (CMB) may be present. To mitigate this risk, both procedural controls and statistical tests were employed. During data collection, we implemented preventive measures through anonymous questionnaires and randomizing item order. Statistically, we conducted a post-hoc assessment using Harman’s single-factor test. The results showed that the variance explained by the first principal component extracted without rotation was 38.5% (less than the 50% threshold), indicating that common method bias was not a significant concern in this study [37].

#### 5.1.2 Reliability analysis.

The reliability of the model was analyzed using Cronbach’s α and composite reliability (CR). As shown in Table 2, both Cronbach’s α and CR values for all variables exceeded 0.7, indicating that the questionnaire possessed good reliability.

**Table 2:** Results of Reliability and convergent validity analysis.

Construct	Items	Factor loading	Cronbach’s α	Composite Reliability	AVE
Relationship Strength	RS1	0.711	0.850	0.854	0.595
	RS2	0.723			
	RS3	0.845			
	RS4	0.798			
Employee Data Literacy	DL1	0.759	0.871	0.872	0.631
	DL2	0.770			
	DL3	0.819			
	DL4	0.828			
Data Quality	DQ1	0.798	0.807	0.814	0.596
	DQ2	0.680			
	DQ3	0.830			
Data Culture	DC1	0.678	0.815	0.821	0.608
	DC2	0.758			
	DC3	0.889			
Institutional Norms	IN1	0.800	0.794	0.805	0.581
	IN2	0.685			
	IN3	0.796			
Data platform integration capability	PI1	0.804	0.804	0.807	0.584
	PI2	0.682			
	PI3	0.800			
Data platform security assurance capability	PS1	0.787	0.798	0.806	0.581
	PS2	0.684			
	PS3	0.810			
Attitude toward Data Sharing	SA1	0.805	0.861	0.866	0.682
	SA2	0.782			
	SA3	0.888			
Data Sharing Intention	SW1	0.897	0.881	0.889	0.728
	SW2	0.780			
	SW3	0.878			
Data Sharing Behavior	SB1	0.630	0.795	0.808	0.587
	SB2	0.807			
	SB3	0.844			

#### 5.1.3 Validity analysis.

Convergent validity was analyzed using confirmatory factor analysis (CFA). As shown in Table 1, the standardized factor loadings of all observed variables exceeded 0.5, the average variance extracted (AVE) values met the reference standard of 0.5, and composite reliability (CR) values exceeded 0.7, confirming the strong convergent validity of the scale.

As shown in Table 3, the square roots of the AVE for each variable were greater than their correlation coefficients with other variables, demonstrating satisfactory discriminant validity.

**Table 3:** Results of Discriminant validity analysis.

	RS	DL	DQ	DC	IN	PI	PS	SA	SW	SB
RS	**0.771**									
DL	0.241	**0.795**								
DQ	0.300	0.273	**0.772**							
DC	0.213	0.336	0.311	**0.780**						
IN	0.156	0.136	0.173	0.231	**0.762**					
PI	0.164	0.215	0.158	0.163	0.136	**0.764**				
PS	0.258	0.177	0.255	0.280	0.228	0.143	**0.762**			
SA	0.444	0.485	0.352	0.453	0.489	0.196	0.271	**0.826**		
SW	0.514	0.513	0.461	0.486	0.391	0.228	0.505	0.626	**0.853**	
SB	0.311	0.292	0.291	0.310	0.252	0.417	0.595	0.360	0.578	**0.766**

### 5.2 Structural model testing

#### 5.2.1 Model fit testing.

**Fig 3:**
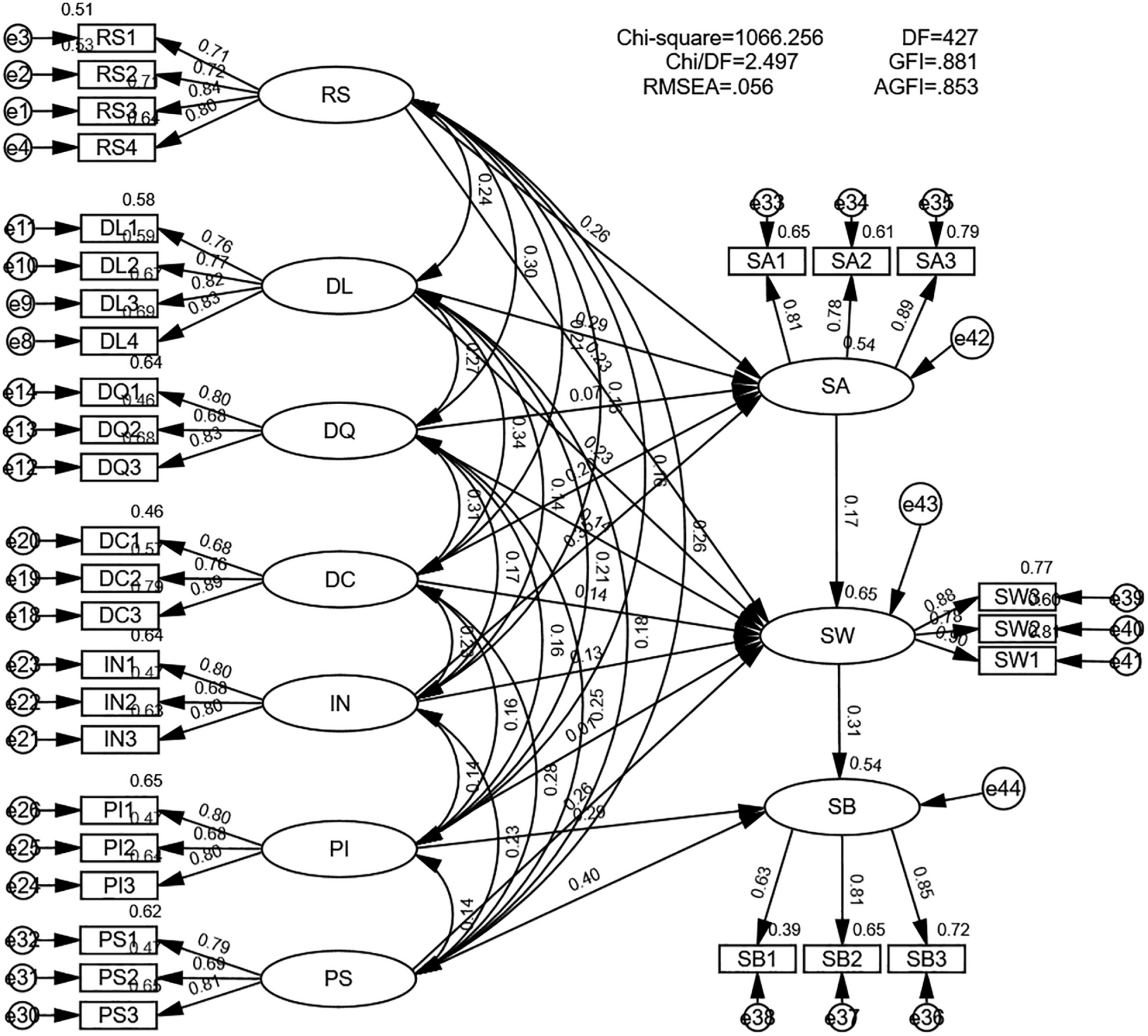
Structural equation model of factors influencing data sharing within CSOEs.

This paper utilized AMOS 28 software to construct a structural equation model and conduct fit testing. The model structure is presented in Fig 3, and the fit results are reported in Table 3.

Following the reviewer’s suggestion to simplify the model, two variables—data standardization and data platform reconfiguration capability—were removed. The results showed that the simplified model exhibited a significant reduction in AIC, achieving a better balance between model fit and parsimony. As shown in Table 4, all indices meet the fit criteria [38,39]. These results demonstrate a strong alignment between the theoretical model and empirical data, supporting the progression to path relationship analysis.

**Table 4:** Results of model fit testing.

	χ^2^/df	RMSEA	SRMR	CFI	TLI	IFI	AGFI	AIC
Original Model	2.180	0.050	0.031	0.923	0.911	0.924	0.848	1591.392
Simplified Model	2.497	0.056	0.031	0.919	0.906	0.920	0.853	1268.256
Recommended value	<5	<0.08	<0.05	>0.90	>0.90	>0.90	>0.80	

We evaluated the explanatory power of the simplified model. As shown in Table 5, the model demonstrated moderate-to-strong explanatory power for the endogenous constructs, including attitude toward data sharing (R^2^ = 0.541), data sharing intention (R^2^ = 0.651), and data sharing behavior (R^2^ = 0.537).

**Table 5:** Explanatory power of endogenous variables.

Endogenous Variable	R^2^	Explanatory Power
Attitude toward Data Sharing	0.541	Moderate
Data Sharing Intention	0.651	Strong
Data Sharing Behavior	0.537	Moderate

#### 5.2.2 Parameter estimation and hypothesis testing.

Based on the theoretical model and sample data, path coefficients were estimated using the maximum likelihood method. Statistical measures, including standardized path coefficients, T-values, and significance levels (p-values), were reported for hypothesis testing. To validate the reliability of the results, the bootstrap method was further employed to test the robustness of the standardized path coefficients. Additionally, the mediating effect of data sharing intention between attitude toward data sharing and data sharing behavior was examined. The results are shown in Table 6.

**Table 6:** Results of hypothesis testing.

Hyp.	Path	Path Coefficient	T Statistics	P Values	Bootstrap (95%)		Conclusion
					Lower	Upper	
H1a	SA < ---RS	0.256	5.626	***	0.164	0.338	Supported
H1b	SW < ---RS	0.225	5.196	***	0.141	0.316	Supported
H2a	SA < ---DL	0.289	6.177	***	0.199	0.375	Supported
H2b	SW < ---DL	0.226	5.079	***	0.129	0.322	Supported
H3a	SA < ---DQ	0.074	1.590	0.112	−0.025	0.169	Not Supported
H3b	SW < ---DQ	0.139	3.324	***	0.055	0.219	Supported
H4a	SA < ---DC	0.197	4.150	***	0.099	0.287	Supported
H4b	SW < ---DC	0.139	3.212	0.001	0.053	0.227	Supported
H5a	SA < ---IN	0.352	7.291	***	0.266	0.446	Supported
H5b	SW < ---IN	0.125	2.757	0.006	0.034	0.224	Supported
H6a	SW < ---PI	0.011	0.280	0.779	0.000	0.000	Not Supported
H6b	SB < ---PI	0.290	6.196	***	0.190	0.403	Supported
H7a	SW < ---PS	0.255	5.978	***	0.154	0.355	Supported
H7b	SB < ---PS	0.396	6.909	***	0.281	0.514	Supported
H8	SW < ---SA	0.172	2.930	0.003	−0.011	0.342	Not Supported
H9	SB < ---SW	0.313	5.923	***	0.151	0.452	Supported
H10	SB < ---SW < ---SA	–	–	–	−0.010	0.135	Not Supported

## 6 Discussion

### 6.1 Key findings and explanations

As shown in Table 6, four hypotheses are invalid, while thirteen are valid. The research results are discussed and analyzed below.

#### 6.1.1 Analysis of influencing factors in the information person dimension.

Both H1a (β = 0.256, p < 0.001) and H1b (β = 0.225, p < 0.001) were supported. This indicates that relationship strength had a significant positive influence on both employees’ attitude toward data sharing and data sharing intention. Previous research indicates that strong relationships promote proactive collaboration and resource sharing among enterprises [40]. Zhang et al. [41] examined business relationships among informal organizations in China, and found that strong relationships could enhance enterprises’ willingness to share resources, thereby improving cooperation efficiency. In the context of cross-organizational data sharing within CSOEs, strong relationships foster trust and reciprocity, reducing transaction costs and perceived risks, and encouraging proactive collaboration among entities. This finding aligns with the conclusions of Kumar et al. [42] and Yang et al. [43] regarding information sharing in supply chains.

Both H2a (β = 0.289, p < 0.001) and H2b (β = 0.226, p < 0.001) were supported, indicating that employee data literacy has a significant positive influence on both attitude and intention. Studies indicate that in the process of enterprise digital transformation, employees’ data literacy is a key variable influencing their behavioral intentions [44]. High data literacy enhances employees’ ability to understand and apply data, thereby increasing their data sharing intention within the organization [45]. Within CSOEs, high data literacy empowers employees with self-efficacy in handling complex data, leading to a more positive attitude toward data sharing and a greater willingness to participate. This finding validates the logic of self-efficacy in Social Cognitive Theory [46].

#### 6.1.2 Analysis of influencing factors in the information resource dimension.

H3a (p > 0.05) was rejected, while H3b (β = 0.139, p < 0.01) was supported. This indicates that data quality was a key factor influencing data sharing intention, but it had no significant effect on attitude. This divergence reveals a modification of the classic technology acceptance logic within a specific institutional context [47].

The support for H3b aligns with common understanding, as multiple studies [48,49] have shown that high-quality data enhance the data sharing intention of individuals or organizations. In the context of cross-organizational data sharing within CSOEs, when data quality is high, employees perceive it as capable of supporting the efficient achievement of business objectives, thereby enhancing their data sharing intention.

However, the rejection of H3a is inconsistent with the expectations of classic theories such as the Technology Acceptance Model (TAM). Salovaara & Tamminen [50] note that the classic TAM treats users as passive technology acceptors, which may be limited in specific organizational contexts. Within CSOEs, characterized by strong administrative directives, organizational normative pressure suppresses the pathway through which technological perceptions shape intrinsic attitudes. This leads to an adaptive reconstruction of the mechanism governing employee attitude formation. Attitudes toward data sharing are no longer primarily determined by technical attributes, but are instead shaped by identification with organizational strategy or commitment to political compliance. Consequently, employees’ attitudes toward data sharing lacked sensitivity to variations in data quality. This finding corroborates the view of Tarhini et al. [51] that a robust theory should fully account for contextual factors such as social and organizational elements.

Specifically, in the context of cross-organizational data sharing within CSOEs, employees’ attitudes toward data sharing manifest as expressions of organizational compliance, while their data sharing intention reflects pragmatic decision-making based on data utility. The separation of data quality’s impact on attitude versus intention is a typical projection of the “semi-administrative, semi-market” dual organizational nature of CSOEs onto individual behavioral decision-making.

#### 6.1.3 Analysis of influencing factors in the information environment dimension.

H4a (β = 0.197, p < 0.001) and H4b (β = 0.139, p < 0.01) were supported, indicating that data culture had a significant positive influence on both attitude and intention.

Social Information Processing Theory [52] posits that individual attitudes and needs are not entirely endogenous, but are constructed by the social environment in which they are situated. As an implicit cognitive environment within the organization, data culture shapes employees’ judgments regarding the value of data sharing. When an enterprise advocates “data as an asset,” employees’ perception of data sharing gradually shifts from resource control to value co-creation, thereby subtly shaping their data sharing awareness. Bock [53] found that a supportive organizational climate can significantly reduce employees’ tendency to hoard knowledge by altering their subjective norms. Similarly, research within the Chinese context indicates that an organizational culture advocating data sharing can foster employees’ data sharing awareness [54].

H5a (β = 0.352, p < 0.001) and H5b (β = 0.125, p < 0.01) were supported, indicating that institutional norms significantly influenced both attitude and intention.

Institutional norms, as an explicit regulatory mechanism within the organization, directly influence employees’ value judgments by establishing behavioral boundaries. Institutional Theory posits that institutions provide a logic of action for organizational members through rule-setting and monitoring mechanisms [55]. Within the bureaucratic structure of CSOEs, institutional norms establish the political legitimacy of data sharing behaviors. Studies in the Chinese context indicate that power distance and collectivist culture reinforce employees’ obedience to organizational norms [56,57]. Essentially, the cognition of data sharing among employees of CSOEs is a rational response to and identification with the organizational regulatory pressures, which is consistent with the strong administrative logic analyzed earlier.

Notably, the impact of institutional norms on attitude (β = 0.352) was significantly stronger than that of data culture (β = 0.197). This indicates that, compared to the subtle influence of culture, the shaping of employee cognition within CSOEs was more sensitive to administrative directives characterized by coercive power and clear reward-punishment orientations.

#### 6.1.4 Analysis of influencing factors in the information technology dimension.

The results reveal distinct mechanisms for “facilitating technology” and “defensive technology” in data sharing within CSOEs.

Data platform integration capability significantly influenced data sharing behavior (β = 0.290, p < 0.001) but had no effect on data sharing intention (p > 0.05). This finding is consistent with the conclusions of Kim et al. [11] in the field of scientific data sharing. In TPB, Ajzen noted that when perceived behavioral control is veridical, it can bypass behavioral intention to directly predict behavior [19]. Similarly, UTAUT posits that facilitating conditions can bypass behavioral intention and directly impact use behavior [58]. As the digital transformation of CSOEs deepens, data sharing is often embedded into unified platforms. High-level integration capability transforms it into structured or even automated processes within business operations. This technology-enabled convenience lowers operational barriers, enabling employees to complete data sharing behaviors while bypassing the decision-making process related to intention. A Chinese study on the digital transformation practice of large enterprises points out that digital platforms provide infrastructural support for business activities, reducing action resistance and enabling behavior to occur naturally [59]. Furthermore, DeSanctis argues that technology itself embodies specific rules and resources, these technical structures interact with organizational social structures, ultimately guiding behavioral patterns [60].

Data platform security assurance capability significantly impacted both intention (β = 0.255, p < 0.001) and behavior (β = 0.396, p < 0.001). Notably, it held the highest impact weight for both intention (β = 0.255) and behavior (β = 0.396). Distinct from data platform integration capability, within the unique ecosystem of CSOEs, security assurance capability transcends mere technical support. It has evolved into a core mechanism for reconstructing organizational trust and balancing risks and benefits, thereby exerting a significant positive influence on both data sharing intention and behavior. In the context of cross-organizational data sharing within CSOEs, data platform security assurance capability acts as a hygiene factor. While platform integration capability addresses the issue of “ease of use,” security assurance capability resolves the trust pain point of “daring to use.” Rebecca [61] showed that perceived security protection significantly enhanced data sharing intention, a finding confirmed by Wen [62].

#### 6.1.5 Analysis of attitude toward data sharing, data sharing intention, and data sharing behavior.

Regarding the transformation mechanism among attitude toward data sharing, data sharing intention, and data sharing behavior, this study presents a challenging finding. Although data sharing intention significantly predicted data sharing behavior (H9 supported), attitude toward data sharing failed to significantly translate into data sharing intention (H8 unsupported). Consequently, the mediating effect of intention between attitude and behavior was not supported (H10 rejected). This implies that in the context of cross-organizational data sharing within CSOEs, the classic TPB exhibits a path adaptive reconstruction, manifesting as a phenomenon of “Cognitive-Intention Decoupling.”

In TPB, the premise for attitude to predict intention is typically that the behavior involves a high degree of autonomy [19]. However, Venkatesh et al. noted that in mandatory or semi-mandatory environments, the influence of Attitude on Intention became non-significant when Performance Expectancy and Effort Expectancy were introduced [58]. Similarly, Brown et al. demonstrated that in mandatory environments, the traditional attitude-intention pathway became altered, with Perceived Utility and Subjective Norm becoming the dominant factors influencing intention [63]. In CSOEs, characterized by strong administrative intervention and high-risk pressure, attitude toward data sharing reflects employees’ value identification with organizational norms. In contrast, data sharing intention is an adaptive reconstruction decision based on pragmatic considerations, contingent upon whether the data solves actual business issues (H3b) and whether adequate security assurance is present (H7a). Consequently, personal affective preferences fail to translate into intentions based on pragmatism. The two constructs follow parallel processing pathways, no longer maintaining a direct causal dependency, leading to the failure of the mediation effect of data sharing intention.

Although attitude toward data sharing fails to directly translate into data sharing intention, once employees form a clear data sharing intention, it will be efficiently converted into actual behavior. This conclusion has been validated in multiple studies [64,65].

Despite the non-significant “attitude-intention” pathway, the research model still demonstrates robust explanatory power. The assessment results show that the variance explanation rates for attitude toward data sharing (R^2^ = 0.541), data sharing intention (R^2^ = 0.651), and data sharing behavior (R^2^ = 0.537) all exceeded 50%. This indicates that the environmental stimuli selected based on Information Ecology Theory effectively explained the formation of employee psychological cognition, and successfully captured the core drivers of data sharing in CSOEs. More importantly, “Cognitive-Intention Decoupling” is not a model flaw; rather, it reflects that within the unique organizational context of CSOEs, data sharing behavior follows a logic of organizational compliance rather than individual rational behavior.

### 6.2 Theoretical implications

By integrating Information Ecology Theory, the SOR framework, and TPB, this study constructs an analytical model for data sharing behavior within CSOEs. The results validate the applicability of this cross-theoretical framework in complex organizational contexts, and reveal the unique logic of data sharing under strong administrative control.

First, this study establishes a data sharing analysis framework adapted to the CSOEs context. By organically integrating Information Ecology Theory, TPB, and the SOR model, a multidimensional framework for analyzing influencing factors was constructed. Information Ecology Theory provides a systematic perspective on these factors, TPB focuses on behavioral driving mechanisms, and the SOR model clarifies the causal chain from environmental stimulus to behavioral response. This framework achieves a systemic integration of the macro-environment, micro-cognition, and behavioral outcomes. It overcomes the explanatory limitations of single theories, providing an interdisciplinary theoretical perspective for explaining data sharing in complex organizations, and offering a reusable analytical tool for future research.

Second, this study defines the boundary conditions of TPB within contexts of strong administrative constraints. While classic TPB posits that attitude is a strong predictor of intention, this study finds that in the context of CSOEs, a positive attitude toward data sharing does not effectively translate into data sharing intention. This finding challenges the universality of mainstream theory, and reveals that the “attitude-intention” pathway within TPB may fail in environments characterized by strong administrative intervention. This offers valuable insights for future research on organizational behavior in high power distance cultures.

Third, this study elucidates the differentiated roles of distinct technical features in driving behavior. The study reveals that facilitating technologies and defensive technologies impact data sharing behavior through distinct mechanisms. The former acts as a “behavioral shortcut,” bypassing data sharing intention to directly promote data sharing behavior. This finding enriches the discussion on facilitating conditions in UTAUT, demonstrating that in specific task contexts, a mature technical environment can directly drive behavior. Meanwhile, defensive technology plays the role of a critical hygiene factor, exerting the strongest influence on both sharing intention and behavior, reflecting the behavioral baseline of security and compliance in strongly regulated and high-risk environments.

### 6.3 Practical implications

Based on the empirical results, this study proposes the following management recommendations.

First, managers should establish a data governance logic characterized by rigid constraints and pragmatism. Given the observed “Cognitive-Intention Decoupling,” managers must recognize that relying solely on cognitive shaping or ideological indoctrination is insufficient to effectively drive data sharing. Instead, the focus should shift to strengthening rigid constraints and pragmatic governance. (1) Reinforce the rigidity of institutional norms. Clearly define the boundaries of rights, responsibilities, and obligations for data sharing within institutional norms, and eliminate gray areas with rigid regulations. Leverage the legitimacy of administrative directives to enhance data sharing intention. (2) Implement technology-enabled behavioral embedding. Seamlessly embed data sharing into business processes, guiding employees to complete data sharing behaviors directly through technical facilitation. (3) Adhere to a utility-oriented approach where usage drives governance. Prioritize the governance of high-frequency application data, and enhance perceived utility through improved data quality, thereby stimulating data sharing intention.

Second, construct a “Technology-Management” integrated trust defense system. On the one hand, increase investment in security technologies such as privacy computing, data encryption, and access control to ensure controllable data flow. On the other hand, combine technical capabilities with managerial exemption mechanisms to reduce employees’ perceived risk of data leakage or non-compliance.

Finally, consolidate the ecological foundation. At the organizational level, establish cross-organizational collaboration mechanisms and normalized communication channels to strengthen inter-organizational relationships. At the individual level, enhance data skills training for all staff to improve their understanding and application of data value. Regarding cultural atmosphere, continuously cultivate data-driven organizational values, and shape an environment supportive of data sharing.

### 6.4 Limitations and future research

This study acknowledges two limitations to be addressed in future research. First, the use of a snowball sampling strategy, while practical given the constraints of obtaining data within CSOEs, may introduce sample selection bias. The sample may be overly concentrated in organizations with higher degrees of digitalization, potentially leading to conclusions that reflect characteristics of an elite group. Future research should employ stratified probability sampling, strictly controlling the distribution of respondents across managerial, technical, and operational roles, to obtain a more representative sample. Second, although the sample covered multiple industries (e.g., energy, infrastructure, and finance), this study conducted a pooled analysis without examining the moderating effects of industry heterogeneity. Future research should further examine specific industry attributes and conduct comparative analyses of data sharing mechanisms across different sectors.

## 7 Conclusion

Existing research on data sharing primarily focuses on single organizational attributes, failing to provide a systematic explanation for data sharing within CSOEs, which are characterized by “semi-administrative, semi-market” dual attributes. To address this gap, this study integrates Information Ecology Theory, the SOR framework, and TPB to construct a model of factors influencing intra-group data sharing in CSOEs. The study yields the following core conclusions:

First, an explanatory framework for data sharing adapted to the complex context of CSOEs was constructed. Through the organic integration of the systematic perspective of Information Ecology Theory, the causal logic of the SOR framework, and the behavioral driving mechanisms of TPB, the empirical results indicate that this model demonstrates excellent adaptability in explaining the data sharing behaviors of employees in CSOEs.

Second, this study identifies a unique phenomenon of “Cognitive-Intention Decoupling” within the CSOEs context. Findings reveal that in organizational environments characterized by strong administrative intervention, employees’ attitudes toward data sharing do not significantly influence their data sharing intention.

Third, the study reveals a mechanism of “Adaptive Reconstruction” regarding attitude under strong administrative directives. Within the bureaucratic structure of CSOEs, organizational normative pressure suppresses the traditional pathway of building attitudes based on technical perceptions. Consequently, data quality has no significant impact on attitude toward data sharing.

Fourth, the study elucidates the differentiated roles of distinct technical features in driving behavior. Data platform integration capability acts as a “behavioral shortcut,” lowering behavioral barriers through technological empowerment. This enables employees to bypass complex intention-related decision-making processes and directly engage in data sharing behavior. Meanwhile, data platform security assurance capability constitutes a critical hygiene factor, serving as the baseline guarantee for stimulating data sharing intention and facilitating data sharing behavior.

It must be acknowledged that due to the specific characteristics of CSOEs such as administrative bureaucracy, political accountability, and strong regulatory pressure, the empirical results of this study may not be directly generalizable to purely market-oriented enterprises. However, they offer valuable structural insights for relevant public sectors or large conglomerate organizations.

## Supporting information

S1 Table Scale for factors influencing data sharing within CSOEs. (DOCX)

S1 Dataset (XLSX)
