# Supplementary material for: Factors influencing intra-group data sharing in China’s central state-owned enterprises: An information ecology perspective
Source: PLoS One. 2026 Feb 20;21(2):e0343558. doi: 10.1371/journal.pone.0343558 (PMC12922980; doi:10.1371/journal.pone.0343558)
Supplement: S1 Table — (DOCX) [file pone.0343558.s001.docx]

| Construct | Items | | Reference | |
| --- | --- | --- | --- | --- |
| **Relationship Strength** | RS1 | Our company frequently communicates with other organizations within the group | (Granovetter, 1973)  (Shi, 2009) | |
|  | RS2 | Our company and other group-internal organizations have a consistent understanding and recognition of data sharing |  |  |
|  | RS3 | Our company receives equal returns from multiple instances of data sharing |  |  |
|  | RS4 | We trust that our counterparts will honor commitments and protect mutual interests |  |  |
| **Employee Data Literacy** | DL1 | I understand the importance of data in empowering business operations | (Serap, 2022) | |
|  | DL2 | I can clearly articulate my data requirements |  |  |
|  | DL3 | I can use data analysis to support my work |  |  |
|  | DL4 | I am aware of the ethical standards and legal requirements for data collection, use, and sharing |  |  |
| **Data Quality** | DQ1 | The data we share accurately describes actual business operations | (Wang, 1996) | |
|  | DQ2 | The data we share is complete |  |  |
|  | DQ3 | The data we share is valid |  |  |
| **Data Culture** | DC1 | Our company has a clear data strategy | (Azeem, 2021) | |
|  | DC2 | Our company actively promotes data-driven decision-making |  |  |
|  | DC3 | Our company regularly conducts training on data application |  |  |
| **Institutional Norms** | IN1 | Our company has clear data sharing policies to guide data sharing activities | (Wang, 2014) | |
|  | IN2 | Our company has established standardized data sharing processes |  |  |
|  | IN3 | Our company has comprehensive data sharing security and privacy protection mechanisms |  |  |
| Platform Integration | PI1 | The data platform has the ability to access data from relevant enterprise information systems within the group | (Cenamor, 2019) | |
|  | PI2 | The data platform has the ability to provide real-time data exchange between enterprises |  |  |
|  | PI3 | The data platform has the ability to extract data directly from databases |  |  |
| Platform Security Assurance | PS1 | The data platform has the ability to ensure data security | (Jiang, 2020) |  |
|  | PS2 | The data platform has the privacy protection capabilities required by regulations |  |  |
|  | PS3 | The data platform can be used with confidence |  |  |
| **Attitude toward Data Sharing** | SA1 | Data sharing is valuable for a company's innovation and development | (Kim, 2018) | |
|  | SA2 | Data sharing is meaningful |  |  |
|  | SA3 | Data sharing is worth promoting |  |  |
| **Data Sharing Intention** | SW1 | If necessary, I am likely to share data | (Kim, 2018) | |
|  | SW2 | If necessary, I intend to share data |  |  |
|  | SW3 | If necessary, I will do my best to share data |  |  |
| **Data Sharing Behavior** | SB1 | I approve of data sharing | (Kim, 2018) |  |
|  | SB2 | I have actively responded to data sharing initiatives |  |  |
|  | SB3 | I will continue to actively respond to data sharing initiatives in the future |  |  |
